# The genome sequence of the acute-angled fungus beetle, *Cryptophagus acutangulus *Gyllenhal, 1827

**DOI:** 10.12688/wellcomeopenres.23688.1

**Published:** 2025-02-10

**Authors:** Maxwell V. L. Barclay, Dmitry Telnov, Natural History Museum Genome Acquisition Lab, Darwin Tree of Life Barcoding collective, Wellcome Sanger Institute Tree of Life Management, Samples and Laboratory team, Wellcome Sanger Institute Scientific Operations: Sequencing Operations, Wellcome Sanger Institute Tree of Life Core Informatics team, Tree of Life Core Informatics collective, Darwin Tree of Life Consortium

**Affiliations:** 1 Natural History Museum, London, England, UK; 2 Daugavpils University, Daugavpils, Latvia; 3 University of Latvia, Rīga, Latvia

**Keywords:** Cryptophagus acutangulus, acute-angled fungus beetle, genome sequence, chromosomal, Coleoptera

## Abstract

We present a genome assembly from an individual male specimen of *Cryptophagus acutangulus* (acute-angled fungus beetle; Arthropoda; Insecta; Coleoptera; Cryptophagidae). The genome sequence has a total length of 177.30 megabases. Most of the assembly (83.62%) is scaffolded into 18 chromosomal pseudomolecules, including the X _1_, X _2_ and Y sex chromosomes. The mitochondrial genome has also been assembled and is 20.2 kilobases in length.

## Species taxonomy

Eukaryota; Opisthokonta; Metazoa; Eumetazoa; Bilateria; Protostomia; Ecdysozoa; Panarthropoda; Arthropoda; Mandibulata; Pancrustacea; Hexapoda; Insecta; Dicondylia; Pterygota; Neoptera; Endopterygota; Coleoptera; Polyphaga; Cucujiformia; Cucujoidea; Cryptophagidae; Cryptophaginae; *Cryptophagus*; *Cryptophagus acutangulus* Gyllenhal, 1827 (NCBI:txid1553241)

## Background

The Cryptophagidae Kirby, 1837, commonly known as silken fungus beetles, is a family of the order Coleoptera with over 600 described extant species worldwide in two subfamilies ( Leschen, 2010). The family is of cosmopolitan distribution with highest diversity in temperate regions. Some silken fungus beetles were considered economically important in the past, for example, as pests of stored products. Most species are associated with decaying organic matter and fungi, adults and larvae are fungus feeding and found in rotting vegetation, social insect and bird nests, flood debris, stored products, dry dung, and similar habitats ( Leschen, 2010 and references therein). The English name of the family refers to the fine, regular setation covering the dorsum of adults. In the British fauna, there are around 100 species of silken fungus beetles known presently ( Duff, 2018), almost all in two large genera, *Cryptophagus* and *Atomaria*. Some are very rare and lacking records for over a half of century ( Duff, 2020), while others may be synathropic introductions that have not survived, or that are maintained by ongoing introductions and do not exist in natural situations. The taxonomy and identification of members of the family are difficult.

The genus *Cryptophagus* Herbst, 1792 is represented in the Palaearctic Region by nearly 150 species ( Esser, 2017a; Esser, 2017b; Esser, 2017c; Johnson *et al.*, 2007; Otero & López, 2011). Approximately eighty species of *Cryptophagus* occur in Europe ( Johnson *et al.*, 2007; Otero & López, 2011), 36 of which comprise the British fauna ( Duff, 2018).

*Cryptophagus acutangulus* Gyllenhal, 1827 is placed in the subfamily Cryptophaginae ( Johnson *et al.*, 2007). Adults of the species are characteristic by their large compound eyes (about two thirds of total head length) with the large facets, the pronotal callus strongly produced laterally and joining the lateral edge of pronotum at an angle. *Cryptophagus acutangulus* is one of the relatively easy species to identify based on external morphology. However, taxonomic confusion in the past has been so great, that in the British fauna ( Duff, 2018) the number of junior synonyms is greater the number of valid species. Undoubtedly taxonomic problems remain, and it is hoped that genomic information will help to clarify the taxonomy of this generally difficult group of beetles.

*Cryptophagus acutangulus* is nearly cosmopolitan in the distribution yet recorded from Europe, Africa, Asia, North and South America but not known from Australia. In the Palaearctic Region, it is widely distributed from Portugal to Japan ( Johnson *et al.*, 2007; Lyubarsky, 2018). Johnson *et al.* (2007) list the species from 31 European countries. Additionally, the species is also known from Bulgaria ( Orlova-Bienkowskaja, 2017), Moldova ( Bacal *et al.*, 2013), and Portugal ( GBIF Secretariat, 2024), but Azerbaijan and Georgia only have a tiny fraction of their land within geographical Europe. Generally, the acute-angled fungus beetle is much less abundant in southern Europe than in the zone of temperate and boreal forests. In Africa it is currently known from the North (Egypt, Morocco), in Asia the species occurs in Arabian Peninsula, northern and northeastern China, Central Asian countries, Korean Peninsula, Japan, Pakistan throughout western and eastern Siberia up to the Russian Far East ( GBIF Secretariat, 2024; Johnson *et al.*, 2007).

*Cryptophagus acutangulus* is in part a synanthropic species. Adults and larvae have been recorded in decaying plant matter (e.g., haystacks), under loose bark, in decaying wood of deciduous and coniferous trees, on sporophores of wood-decay fungi such as *Pleurotus* spp., *Cerioporus squamosus* (Huds.) Quélet (1886), as well as on *Penicillus* spp. and *Trichoderma* spp. microscopic fungi and in warehouses ( Duff, 2020; Koch, 1992; Nikitsky *et al.*, 1996). The species is described as eurytopic, partially synanthropic, phytodetriticol and mycetophagous ( Koch, 1992). Adult beetles in Britain are found in vegetable litter, haystacks, mouldy plant material and in warehouses ( Duff, 2020) as well as on flowers. *Cryptophagus acutangulus* larvae and adults have been found associated with carrion in caves ( Macdonald, 1992).

In the British Isles adults of *Cryptophagus acutangulus* have been reported all year ( Duff, 2020). It is widespread but uncommon across England and Wales, with old data from Scotland, and is known in Ireland ( Duff, 2020). The species was not listed in the national Red Data Book ( Shirt, 1987) and has no conservation status in the United Kingdom.

**Figure 1.**
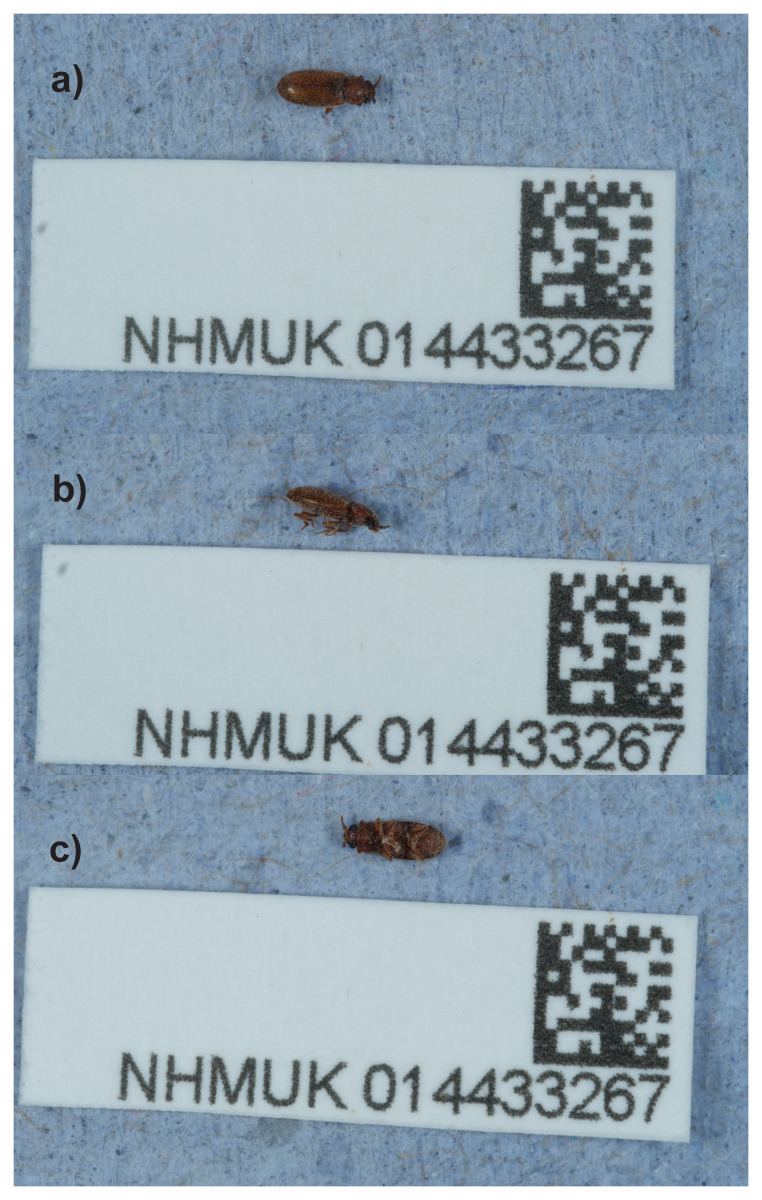
Photographs of the *Cryptophagus acutangulus* (icCryAcut1) specimen used for genome sequencing.

The adult specimen used for sequencing ( Figure 1) was obtained in Fulham, London, England by beating blossoming trees in an urban setting on 2021-05-24, collected and identified by M.V.L. Barclay. There were compost heaps and pigeon nests close by, which may have provided larval habitat.

## Genome sequence report

### Sequencing data

The genome of a specimen of *Cryptophagus acutangulus* ( Figure 1) was sequenced using Pacific Biosciences single-molecule HiFi long reads, generating 27.10 Gb from 2.75 million reads. GenomeScope analysis of the PacBio HiFi data estimated the haploid genome size at 166.07 Mb, with a heterozygosity of 1.91% and repeat content of 47.35%. These values provide an initial assessment of genome complexity and the challenges anticipated during assembly. Based on this estimated genome size, the sequencing data provided approximately 141.0x coverage of the genome. Chromosome conformation Hi-C sequencing produced 106.50 Gb from 705.27 million reads.

Table 1 summarises the specimen and sequencing information, including the BioProject, study name, BioSample numbers, and sequencing data for each technology.

**Table 1.** Specimen and sequencing data for *Cryptophagus acutangulus*.

Project information			
**Study title**	Cryptophagus acutangulus		
**Umbrella BioProject**	PRJEB58934		
**Species**	*Cryptophagus acutangulus*		
**BioSpecimen**	SAMEA14448405		
**NCBI taxonomy ID**	1553241		
Specimen information			
**Technology**	**ToLID**	**BioSample** **accession**	**Organism part**
**PacBio long read sequencing**	icCryAcut1	SAMEA14448776	abdomen
**Hi-C sequencing**	icCryAcut1	SAMEA14448775	head and thorax
Sequencing information			
**Platform**	**Run accession**	**Read count**	**Base count (Gb)**
**Hi-C Illumina NovaSeq 6000**	ERR10786008	7.05e+08	106.5
**PacBio Sequel IIe**	ERR10798422	2.75e+06	27.1

***Assembly statistics***

The primary haplotype was assembled, and contigs corresponding to an alternate haplotype were also deposited in INSDC databases. The assembly was improved by manual curation, which corrected 30 misjoins or missing joins and removed four haplotypic duplications. These interventions reduced the total assembly length by 2.4% and decreased the scaffold count by 3.4%. The final assembly has a total length of 177.32 Mb in 368 scaffolds, with 540 gaps, and a scaffold N50 of 9.22 Mb ( Table 2).

**Table 2.** Genome assembly data for *Cryptophagus acutangulus*.

Genome assembly		
Assembly name	icCryAcut1.1	
Assembly accession	GCA_963556235.1	
*Alternate haplotype accession*	*GCA_963556225.1*	
Assembly level for primary assembly	chromosome	
Span (Mb)	177.32	
Number of contigs	908	
Number of scaffolds	368	
Longest scaffold (Mb)	15.64	
Assembly metric	Measure	*Benchmark*
Contig N50 length	0.62 Mb	*≥ 1 Mb*
Scaffold N50 length	9.22 Mb	*= chromosome N50*
Consensus quality (QV)	Primary: 57.5; alternate: 60.5; combined 58.6	*≥ 40*
*k*-mer completeness	Primary: 77.00%; alternate: 61.92%; combined: 96.91%	*≥ 95%*
BUSCO *	C:98.4%[S:97.6%,D:0.8%], F:0.1%,M:1.5%,n:2,124	*S > 90%; D < 5%*
Percentage of assembly mapped to chromosomes	83.43%	*≥ 90%*
Sex chromosomes	X _1_, X _2_, and Y	*localised homologous pairs*
Organelles	Mitochondrial genome: 20.2 kb	*complete single alleles*

* BUSCO scores based on the endopterygota_odb10 BUSCO set using version 5.4.3. C = complete [S = single copy, D = duplicated], F = fragmented, M = missing, n = number of orthologues in comparison.

**Figure 2.**
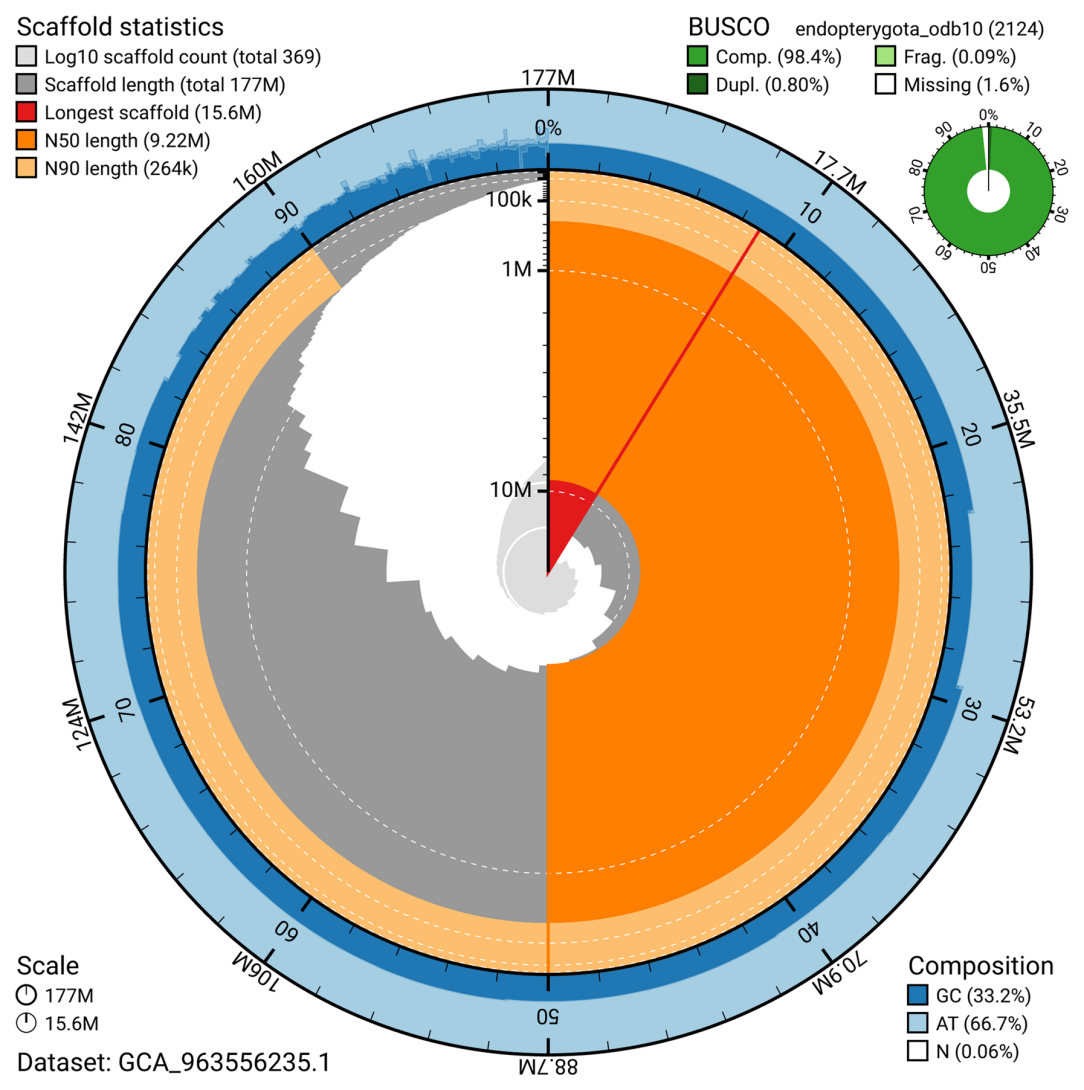
Genome assembly of *Cryptophagus acutangulus*, icCryAcut1.1: metrics. The BlobToolKit snail plot provides an overview of assembly metrics and BUSCO gene completeness. The circumference represents the length of the whole genome sequence, and the main plot is divided into 1,000 bins around the circumference. The outermost blue tracks display the distribution of GC, AT, and N percentages across the bins. Scaffolds are arranged clockwise from longest to shortest and are depicted in dark grey. The longest scaffold is indicated by the red arc, and the deeper orange and pale orange arcs represent the N50 and N90 lengths. A light grey spiral at the centre shows the cumulative scaffold count on a logarithmic scale. A summary of complete, fragmented, duplicated, and missing BUSCO genes in the endopterygota_odb10 set is presented at the top right. An interactive version of this figure is available at https://blobtoolkit.genomehubs.org/view/GCA_963556235.1/dataset/GCA_963556235.1/snail.

**Figure 3.**
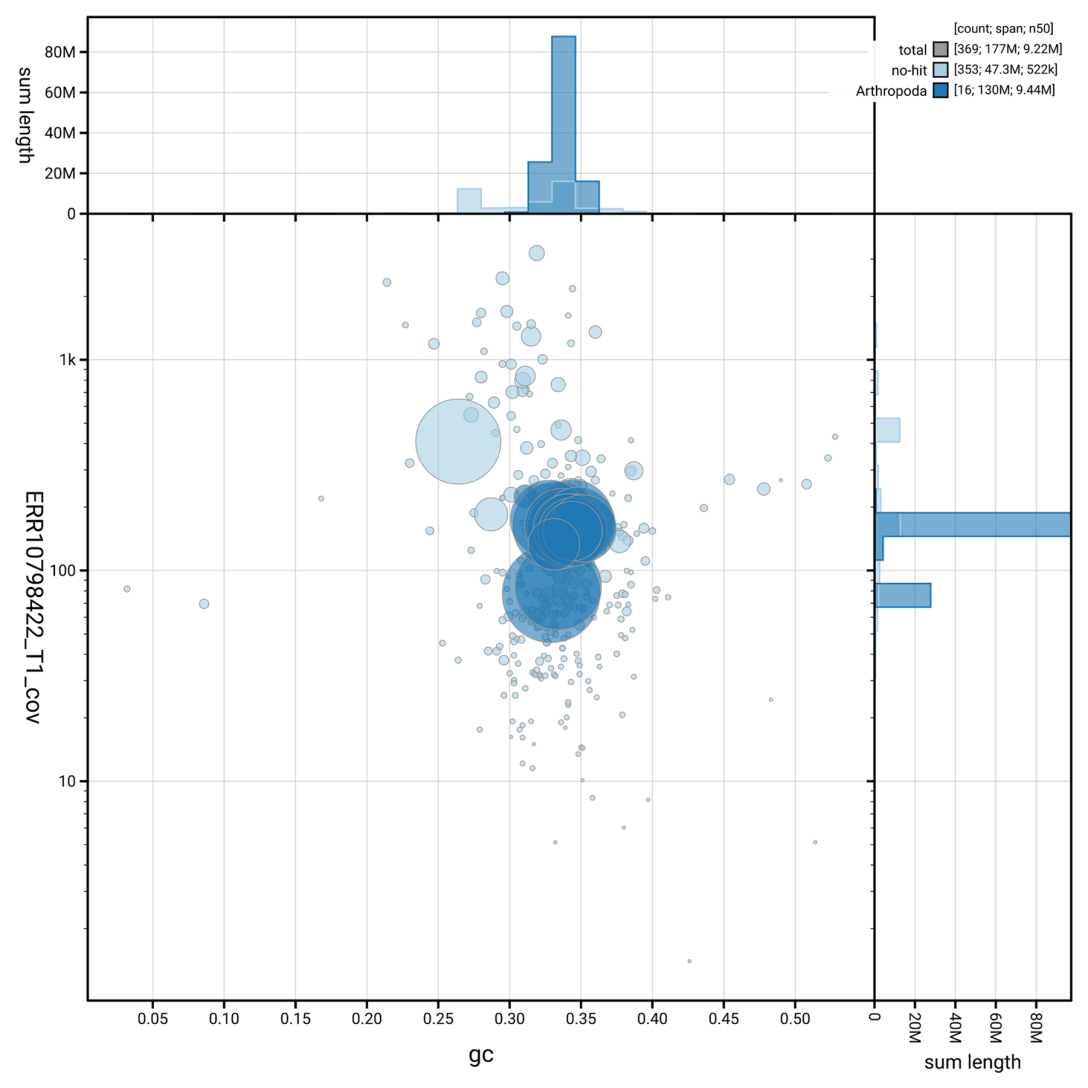
Genome assembly of *Cryptophagus acutangulus*, icCryAcut1.1: BlobToolKit GC-coverage plot showing sequence coverage (vertical axis) and GC content (horizontal axis). The circles represent scaffolds, with the size proportional to scaffold length and the colour representing phylum membership. The histograms along the axes display the total length of sequences distributed across different levels of coverage and GC content. An interactive version of this figure is available at https://blobtoolkit.genomehubs.org/view/GCA_963556235.1/dataset/GCA_963556235.1/blob.

**Figure 4.**
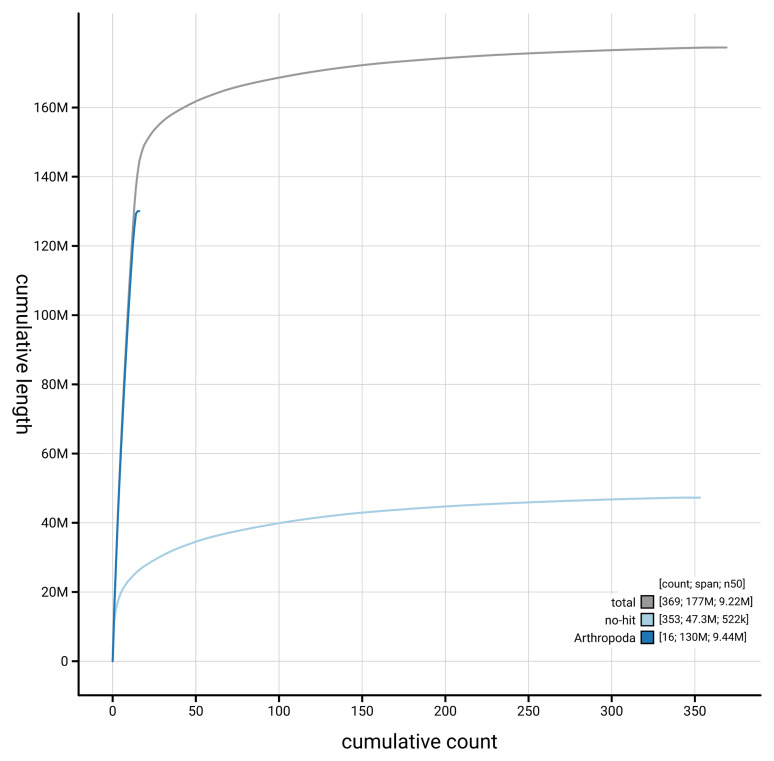
Genome assembly of *Cryptophagus acutangulus* icCryAcut1.1: BlobToolKit cumulative sequence plot. The grey line shows cumulative length for all scaffolds. Coloured lines show cumulative lengths of scaffolds assigned to each phylum using the buscogenes taxrule. An interactive version of this figure is available at https://blobtoolkit.genomehubs.org/view/GCA_963556235.1/dataset/GCA_963556235.1/cumulative.

The snail plot in Figure 2 provides a summary of the assembly statistics, indicating the distribution of scaffold lengths and other assembly metrics. Figure 3 shows the distribution of scaffolds by GC proportion and coverage. Figure 4 presents a cumulative assembly plot, with separate curves representing different scaffold subsets assigned to various phyla, illustrating the completeness of the assembly.

**Figure 5.**
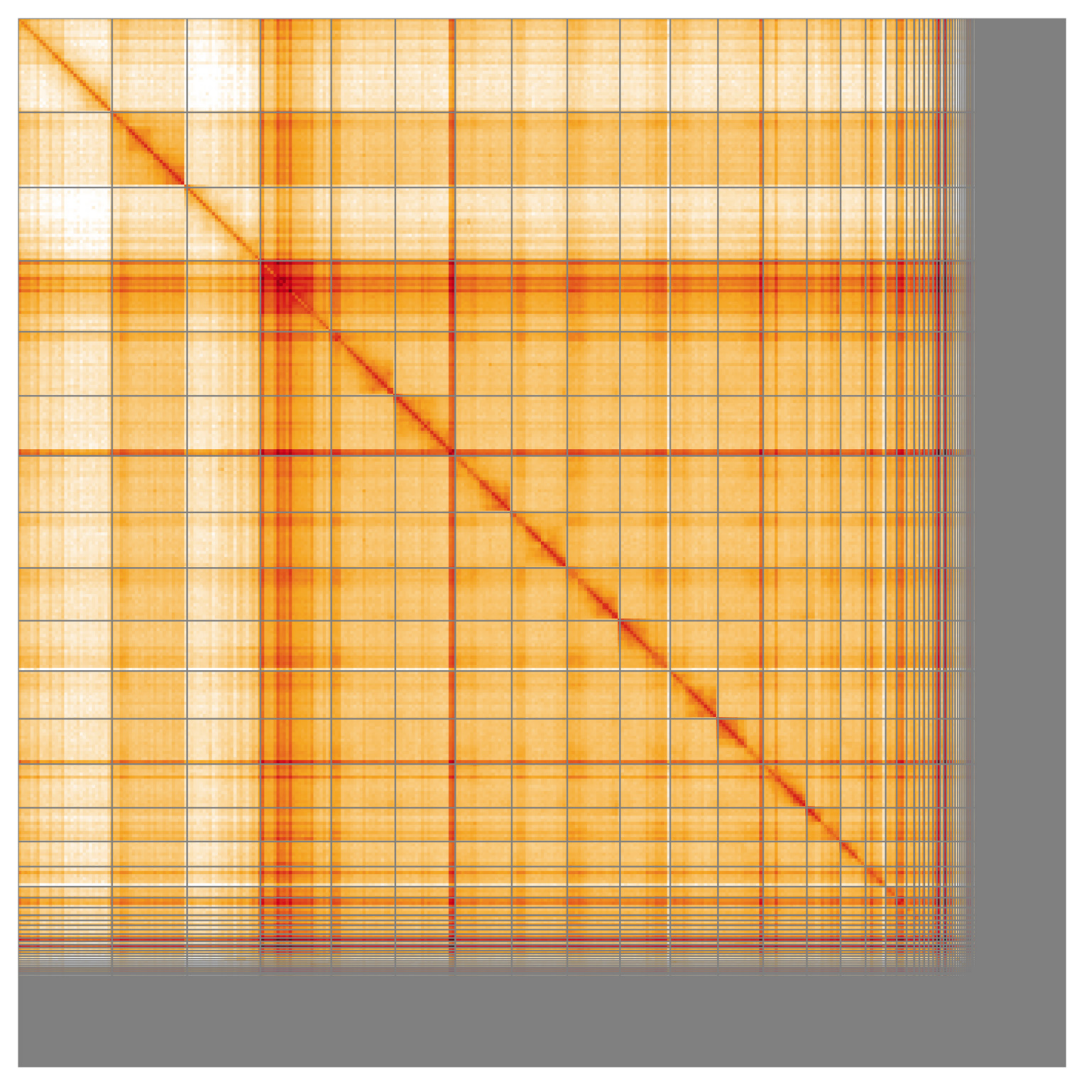
Genome assembly of *Cryptophagus acutangulus* icCryAcut1.1: Hi-C contact map of the icCryAcut1.1 assembly, visualised using HiGlass. Chromosomes are shown in order of size from left to right and top to bottom. An interactive version of this figure may be viewed at https://genome-note-higlass.tol.sanger.ac.uk/l/?d=AmbaYBtLTuWjntuDQJaaGQ.

Most of the assembly sequence (83.43%) was assigned to 18 chromosomal-level scaffolds, representing 15 autosomes and the X _1_, X _2_, and Y sex chromosomes. These chromosome-level scaffolds, confirmed by Hi-C data, are named according to size ( Figure 5; Table 3). During curation, chromosomes X _1_, X _2_ and Y were determined by synteny to the genome of *Brachypterus glaber* (GCA_958510825.1).

**Table 3.** Chromosomal pseudomolecules in the genome assembly of *Cryptophagus acutangulus*, icCryAcut1.

INSDC accession	Name	Length (Mb)	GC%
OY744559.1	1	12.48	33.5
OY744560.1	2	11.78	26.5
OY744561.1	3	10.68	34.5
OY744562.1	4	9.96	33
OY744563.1	5	9.44	34
OY744564.1	6	9.22	33.5
OY744565.1	7	8.78	35
OY744566.1	8	8.37	34.5
OY744567.1	9	7.95	34
OY744568.1	10	7.54	34.5
OY744569.1	11	7.24	35
OY744570.1	12	5.62	34.5
OY744571.1	13	4.16	33
OY744572.1	14	3.35	34
OY744573.1	15	1.84	34
OY744574.1	Y	1.64	28.5
OY744575.1	MT	0.02	23
OY744557.1	X1	15.64	33
OY744558.1	X2	12.2	33.5

The mitochondrial genome was also assembled. This sequence is included as a contig in the multifasta file of the genome submission and as a standalone record in GenBank.

***Assembly quality metrics***

The estimated Quality Value (QV) and *k*-mer completeness metrics, along with BUSCO completeness scores, were calculated for each haplotype and the combined assembly. The QV reflects the base-level accuracy of the assembly, while *k*-mer completeness indicates the proportion of expected *k*-mers identified in the assembly. BUSCO scores provide a measure of completeness based on benchmarking universal single-copy orthologues.

The primary haplotype has a QV of 57.5, and the combined primary and alternate assemblies achieve an estimated QV of 58.6. The *k*-mer completeness for the primary haplotype is 77.00%, and for the alternate haplotype it is 61.92%. The combined primary and alternate assemblies achieve a *k*-mer completeness of 96.91%. BUSCO analysis using the endopterygota_odb10 reference set ( *n* = 2,124) indicated a completeness score of 98.4% (single = 97.6%, duplicated = 0.8%).

Table 2 provides assembly metric benchmarks adapted from Rhie *et al.* (2021) and the Earth BioGenome Project Report on Assembly Standards September 2024. The assembly achieves the EBP reference standard of 5.6.57.

## Methods

### Sample acquisition and DNA barcoding

An adult male *Cryptophagus acutangulus* (specimen ID NHMUK014433267, ToLID icCryAcut1) was collected from Fulmead Street, Parsons Green, England, United Kingdom (latitude 51.47, longitude -0.18) on 2021-05-24 by handpicking. The specimen was collected and identified by Maxwell Barclay (Natural History Museum) and preserved by dry freezing (–80 °C).

The initial identification by morphology was verified by an additional DNA barcoding process according to the framework developed by Twyford *et al*. (2024). A small sample was dissected from the specimen and stored in ethanol, while the remaining parts were shipped on dry ice to the Wellcome Sanger Institute (WSI) ( Pereira *et al.*, 2022). The tissue was lysed, the COI marker region was amplified by PCR, and amplicons were sequenced and compared to the BOLD database, confirming the species identification ( Crowley *et al.*, 2023). Following whole genome sequence generation, the relevant DNA barcode region was also used alongside the initial barcoding data for sample tracking at the WSI ( Twyford *et al.*, 2024). The standard operating procedures for Darwin Tree of Life barcoding have been deposited on protocols.io ( Beasley *et al.*, 2023).

Metadata collection for samples adhered to the Darwin Tree of Life project standards described by Lawniczak *et al*. (2022).

### Nucleic acid extraction

The workflow for high molecular weight (HMW) DNA extraction at the Wellcome Sanger Institute (WSI) Tree of Life Core Laboratory includes a sequence of procedures: sample preparation and homogenisation, DNA extraction, fragmentation and purification. Detailed protocols are available on protocols.io ( Denton *et al.*, 2023b).

The icCryAcut1 sample was prepared for DNA extraction by weighing and dissecting it on dry ice ( Jay *et al.*, 2023). Tissue from the abdomen was homogenised using a PowerMasher II tissue disruptor ( Denton *et al.*, 2023a). HMW DNA was extracted using the Automated MagAttract v1 protocol ( Sheerin *et al.*, 2023). Sheared DNA was purified by solid-phase reversible immobilisation, using AMPure PB beads to eliminate shorter fragments and concentrate the DNA ( Strickland *et al.*, 2023). The concentration of the sheared and purified DNA was assessed using a Nanodrop spectrophotometer and a Qubit Fluorometer using the Qubit dsDNA High Sensitivity Assay kit. The fragment size distribution was evaluated by running the sample on the FemtoPulse system.

### Hi-C sample preparation

Tissue from the head and thorax of the icCryAcut1 sample was processed for Hi-C sequencing at the WSI Scientific Operations core, using the Arima-HiC v2 kit. In brief, 20–50 mg of frozen tissue (stored at –80 °C) was fixed, and the DNA crosslinked using a TC buffer with 22% formaldehyde concentration. After crosslinking, the tissue was homogenised using the Diagnocine Power Masher-II and BioMasher-II tubes and pestles. Following the Arima-HiC v2 kit manufacturer's instructions, crosslinked DNA was digested using a restriction enzyme master mix. The 5’-overhangs were filled in and labelled with biotinylated nucleotides and proximally ligated. An overnight incubation was carried out for enzymes to digest remaining proteins and for crosslinks to reverse. A clean up was performed with SPRIselect beads prior to library preparation. Additionally, the biotinylation percentage was estimated using the Qubit Fluorometer v4.0 (Thermo Fisher Scientific) and Qubit HS Assay Kit and Arima-HiC v2 QC beads.

### Library preparation and sequencing

Library preparation and sequencing were performed at the WSI Scientific Operations core.

***PacBio HiFi (ULI)***

The sample requires Covaris g-TUBE shearing to approximately 10 kb prior to library preparation. Ultra-low input libraries were prepared using PacBio SMRTbell® Express Template Prep Kit 2.0 and PacBio SMRTbell® gDNA Sample Amplification Kit. To begin, samples were normalised to 20 ng of DNA. Initial removal of single-strand overhangs, DNA damage repair, and end repair/A-tailing were performed per manufacturer’s instructions. From the SMRTbell® gDNA Sample Amplification Kit, amplification adapters were then ligated. A 0.85X pre-PCR clean-up was performed with Promega ProNex beads and the sample was then divided into two for a dual PCR. PCR reactions A and B each followed the PCR programs as described in the manufacturer’s protocol. A 0.85X post-PCR clean-up was performed with ProNex beads for PCR reactions A and B and DNA concentration was quantified using the Qubit Fluorometer v4.0 (Thermo Fisher Scientific) and Qubit HS Assay Kit and fragment size analysis was carried out using the Agilent Femto Pulse Automated Pulsed Field CE Instrument (Agilent Technologies) and gDNA 55 kb BAC analysis kit. PCR reactions A and B were then pooled, ensuring the total mass was ≥500 ng in 47.4 μl. The pooled sample then repeated the process for DNA damage repair, end repair/A-tailing and additional hairpin adapter ligation. A 1X clean-up was performed with ProNex beads and DNA concentration was quantified using the Qubit and fragment size analysis was carried out using the Agilent Femto Pulse Automated Pulsed Field CE Instrument (Agilent Technologies). Size selection was performed using Sage Sciences' PippinHT system with target fragment size determined by analysis from the Femto Pulse, usually a value between 4000 and 9000 bp. Size selected libraries were then cleaned-up using1.0X ProNex beads and normalised to 2 nM before proceeding to sequencing.

Samples were sequenced using the Sequel IIe system (Pacific Biosciences, California, USA). The concentration of the library loaded onto the Sequel IIe was in the range 40–135 pM. The SMRT link software, a PacBio web-based end-to-end workflow manager, was used to set-up and monitor the run, as well as perform primary and secondary analysis of the data upon completion.

***Hi-C***

For Hi-C library preparation, DNA was fragmented using the Covaris E220 sonicator (Covaris) and size selected using SPRISelect beads to 400 to 600 bp. The DNA was then enriched using the Arima-HiC v2 kit Enrichment beads. Using the NEBNext Ultra II DNA Library Prep Kit (New England Biolabs) for end repair, a-tailing, and adapter ligation. This uses a custom protocol which resembles the standard NEBNext Ultra II DNA Library Prep protocol but where library preparation occurs while DNA is bound to the Enrichment beads. For library amplification, 10 to 16 PCR cycles were required, determined by the sample biotinylation percentage. The Hi-C sequencing was performed using paired-end sequencing with a read length of 150 bp on an Illumina NovaSeq 6000 instrument.

### Genome assembly, curation and evaluation

***Assembly***

Prior to assembly of the PacBio HiFi reads, a database of *k*-mer counts ( *k* = 31) was generated from the filtered reads using FastK. GenomeScope2 ( Ranallo-Benavidez *et al.*, 2020) was used to analyse the *k*-mer frequency distributions, providing estimates of genome size, heterozygosity, and repeat content.

The HiFi reads were first assembled using Hifiasm ( Cheng *et al.*, 2021) with the --primary option. Haplotypic duplications were identified and removed using purge_dups ( Guan *et al.*, 2020). The Hi-C reads were mapped to the primary contigs using bwa-mem2 ( Vasimuddin *et al.*, 2019). The contigs were further scaffolded using the provided Hi-C data ( Rao *et al.*, 2014) in YaHS ( Zhou *et al.*, 2023) using the --break option for handling potential misassemblies. The scaffolded assemblies were evaluated using Gfastats ( Formenti *et al.*, 2022), BUSCO ( Manni *et al.*, 2021) and MERQURY.FK ( Rhie *et al.*, 2020).

The mitochondrial genome was assembled using MitoHiFi ( Uliano-Silva *et al.*, 2023), which runs MitoFinder ( Allio *et al.*, 2020) and uses these annotations to select the final mitochondrial contig and to ensure the general quality of the sequence.

***Assembly curation***

The assembly was decontaminated using the Assembly Screen for Cobionts and Contaminants (ASCC) pipeline (article in preparation). Flat files and maps used in curation were generated in TreeVal ( Pointon *et al.*, 2023). Manual curation was primarily conducted using PretextView ( Harry, 2022), with additional insights provided by JBrowse2 ( Diesh *et al.*, 2023) and HiGlass ( Kerpedjiev *et al.*, 2018). Scaffolds were visually inspected and corrected as described by Howe *et al*. (2021). Any identified contamination, missed joins, and mis-joins were corrected, and duplicate sequences were tagged and removed. Sex chromosomes were identified by {synteny analysis. The curation process is documented at https://gitlab.com/wtsi-grit/rapid-curation (article in preparation).

***Assembly quality assessment***

The Merqury.FK tool ( Rhie *et al.*, 2020), run in a Singularity container ( Kurtzer *et al.*, 2017), was used to evaluate *k*-mer completeness and assembly quality for the primary and alternate haplotypes using the *k*-mer databases ( *k* = 31) that were computed prior to genome assembly. The analysis outputs included assembly QV scores and completeness statistics.

A Hi-C contact map was produced for the final version of the assembly. The Hi-C reads were aligned using bwa-mem2 ( Vasimuddin *et al.*, 2019) and the alignment files were combined using SAMtools ( Danecek *et al.*, 2021). The Hi-C alignments were converted into a contact map using BEDTools ( Quinlan & Hall, 2010) and the Cooler tool suite ( Abdennur & Mirny, 2020). The contact map is visualised in HiGlass ( Kerpedjiev *et al.*, 2018).

The blobtoolkit pipeline is a Nextflow port of the previous Snakemake Blobtoolkit pipeline ( Challis *et al.*, 2020). It aligns the PacBio reads in SAMtools and minimap2 ( Li, 2018) and generates coverage tracks for regions of fixed size. In parallel, it queries the GoaT database ( Challis *et al.*, 2023) to identify all matching BUSCO lineages to run BUSCO ( Manni *et al.*, 2021). For the three domain-level BUSCO lineages, the pipeline aligns the BUSCO genes to the UniProt Reference Proteomes database ( Bateman *et al.*, 2023) with DIAMOND blastp ( Buchfink *et al.*, 2021). The genome is also divided into chunks according to the density of the BUSCO genes from the closest taxonomic lineage, and each chunk is aligned to the UniProt Reference Proteomes database using DIAMOND blastx. Genome sequences without a hit are chunked using seqtk and aligned to the NT database with blastn ( Altschul *et al.*, 1990). The blobtools suite combines all these outputs into a blobdir for visualisation.

The blobtoolkit pipeline was developed using nf-core tooling ( Ewels *et al.*, 2020) and MultiQC ( Ewels *et al.*, 2016), relying on the Conda package manager, the Bioconda initiative ( Grüning *et al.*, 2018), the Biocontainers infrastructure ( da Veiga Leprevost *et al.*, 2017), as well as the Docker ( Merkel, 2014) and Singularity ( Kurtzer *et al.*, 2017) containerisation solutions.

Table 4 contains a list of relevant software tool versions and sources.

**Table 4.** Software tools: versions and sources.

Software tool	Version	Source
BEDTools	2.30.0	https://github.com/arq5x/bedtools2
BLAST	2.14.0	ftp://ftp.ncbi.nlm.nih.gov/blast/executables/blast+/
BlobToolKit	4.3.9	https://github.com/blobtoolkit/blobtoolkit
BUSCO	5.5.0	https://gitlab.com/ezlab/busco
bwa-mem2	2.2.1	https://github.com/bwa-mem2/bwa-mem2
Cooler	0.8.11	https://github.com/open2c/cooler
DIAMOND	2.1.8	https://github.com/bbuchfink/diamond
fasta_windows	0.2.4	https://github.com/tolkit/fasta_windows
FastK	427104ea91c78c3b8b8b49f1a7d6bbeaa869ba1c	https://github.com/thegenemyers/FASTK
Gfastats	1.3.6	https://github.com/vgl-hub/gfastats
GoaT CLI	0.2.5	https://github.com/genomehubs/goat-cli
Hifiasm	0.16.1-r375	https://github.com/chhylp123/hifiasm
HiGlass	44086069ee7d4d3f6f3f0012569789ec138f42b84a a44357826c0b6753eb28de	https://github.com/higlass/higlass
MerquryFK	d00d98157618f4e8d1a9190026b19b471055b22e	https://github.com/thegenemyers/MERQURY.FK
Minimap2	2.24-r1122	https://github.com/lh3/minimap2
MitoHiFi	2	https://github.com/marcelauliano/MitoHiFi
MultiQC	1.14, 1.17, and 1.18	https://github.com/MultiQC/MultiQC
NCBI Datasets	15.12.0	https://github.com/ncbi/datasets
Nextflow	23.04.1	https://github.com/nextflow-io/nextflow
PretextView	0.2.5	https://github.com/sanger-tol/PretextView
purge_dups	1.2.3	https://github.com/dfguan/purge_dups
samtools	1.19.2	https://github.com/samtools/samtools
sanger-tol/ascc	-	https://github.com/sanger-tol/ascc
sanger-tol/blobtoolkit	0.5.1	https://github.com/sanger-tol/blobtoolkit
Seqtk	1.3	https://github.com/lh3/seqtk
Singularity	3.9.0	https://github.com/sylabs/singularity
TreeVal	1.2.0	https://github.com/sanger-tol/treeval
YaHS	yahs-1.2a.2	https://github.com/c-zhou/yahs

### Wellcome Sanger Institute – Legal and Governance

The materials that have contributed to this genome note have been supplied by a Darwin Tree of Life Partner. The submission of materials by a Darwin Tree of Life Partner is subject to the **‘Darwin Tree of Life Project Sampling Code of Practice’**, which can be found in full on the Darwin Tree of Life website here. By agreeing with and signing up to the Sampling Code of Practice, the Darwin Tree of Life Partner agrees they will meet the legal and ethical requirements and standards set out within this document in respect of all samples acquired for, and supplied to, the Darwin Tree of Life Project.

Further, the Wellcome Sanger Institute employs a process whereby due diligence is carried out proportionate to the nature of the materials themselves, and the circumstances under which they have been/are to be collected and provided for use. The purpose of this is to address and mitigate any potential legal and/or ethical implications of receipt and use of the materials as part of the research project, and to ensure that in doing so we align with best practice wherever possible. The overarching areas of consideration are:

•   Ethical review of provenance and sourcing of the material

•   Legality of collection, transfer and use (national and international)

Each transfer of samples is further undertaken according to a Research Collaboration Agreement or Material Transfer Agreement entered into by the Darwin Tree of Life Partner, Genome Research Limited (operating as the Wellcome Sanger Institute), and in some circumstances other Darwin Tree of Life collaborators.

## Data Availability

European Nucleotide Archive: Cryptophagus acutangulus. Accession number PRJEB58934; https://identifiers.org/ena.embl/PRJEB58934. The genome sequence is released openly for reuse. The *Cryptophagus acutangulus* genome sequencing initiative is part of the Darwin Tree of Life (DToL) project. All raw sequence data and the assembly have been deposited in INSDC databases. The genome will be annotated using available RNA-Seq data and presented through the Ensembl pipeline at the European Bioinformatics Institute. Raw data and assembly accession identifiers are reported in Table 1 and Table 2.

## Author information

Members of the Natural History Museum Genome Acquisition Lab are listed here: https://doi.org/10.5281/zenodo.12159242.

Members of the Darwin Tree of Life Barcoding collective are listed here: https://doi.org/10.5281/zenodo.12158331.

Members of the Wellcome Sanger Institute Tree of Life Management, Samples and Laboratory team are listed here: https://doi.org/10.5281/zenodo.12162482.

Members of Wellcome Sanger Institute Scientific Operations: Sequencing Operations are listed here: https://doi.org/10.5281/zenodo.12165051.

Members of the Wellcome Sanger Institute Tree of Life Core Informatics team are listed here: https://doi.org/10.5281/zenodo.12160324.

Members of the Tree of Life Core Informatics collective are listed here: https://doi.org/10.5281/zenodo.12205391.

Members of the Darwin Tree of Life Consortium are listed here: https://doi.org/10.5281/zenodo.4783558.
