# Supplementary material for: The genome sequence of the acute-angled fungus beetle, Cryptophagus acutangulus Gyllenhal, 1827
Source: Wellcome Open Res. 2025 Feb 10;10:58. [Version 1] doi: 10.12688/wellcomeopenres.23688.1 (PMC12381558; doi:10.12688/wellcomeopenres.23688.1)
Supplement: Peer review history

# Reviewer report: Reviewer response for version 1

**DOI:** 10.21956/wellcomeopenres.26133.r127270

**Published:** 2025-08-29

**Authors:** Terrence Sylvester

**Affiliations:** 1 The University of Memphis, Memphis, Tennessee, USA

The authors present the genome sequence of the acute-angled fungus beetle, *Cryptophagus acutangulus*. The manuscript begins with an adequate background section covering the species’ distribution, taxonomy, and morphology, which is suitable for the scope of this report. The sequencing effort has generated a large volume of data with exceptional depth of coverage, and the authors have used the raw reads to estimate genome size and heterozygosity, providing an initial assessment of genome complexity. The assembly statistics and quality matrices indicate a high-quality reference genome, which will be a valuable resource for comparative studies on beetle and insect genome evolution.

 The authors report a complex chromosome system (XXY) for this species. While full validation may be outside the scope of the current report, confirming these findings through cytogenetic methods would significantly strengthen the conclusion. Furthermore, it would be useful to discuss whether the two X chromosomes arose from an X chromosome fission event or from a fusion between an autosome and the Y chromosome. Adding chromosome labels to the contact matrix would also improve interpretability for readers.

Are sufficient details of methods and materials provided to allow replication by others?

Yes

Is the rationale for creating the dataset(s) clearly described?

Yes

Are the datasets clearly presented in a useable and accessible format?

Yes

Are the protocols appropriate and is the work technically sound?

Yes

Reviewer Expertise:

Genomics

I confirm that I have read this submission and believe that I have an appropriate level of expertise to confirm that it is of an acceptable scientific standard.

# Reviewer report: Reviewer response for version 1

**DOI:** 10.21956/wellcomeopenres.26133.r127272

**Published:** 2025-08-26

**Authors:** Nadège Guiglielmoni

**Affiliations:** 1 University of Cologne, Köln, Germany

The authors present a chromosome-level assembly of the acute-angled fungus beetle Cryptophagus acutangulus which includes sexual chromosomes. The assembly, obtained using ULI PacBio HiFi and Hi-C reads from a single male individual, has 18 chromosome candidates and a good completeness.

 The results and methods are generally satisfactory. I am a bit curious about k-mer spectra. The metrics given by GenomeScope are detailed in the text but I did not see the plot. The k-mer completeness may also seem not ideal, and I imagine it might be that the k-mer plot is not so smooth because of the ULI protocol. But the plot is not there to assess this.

 Also, for the HMW extractions, it is mentioned that the DNA was checked with Nanodrop. If the authors still have the OD values, I would suggest to add them, so that other researchers attempting to apply these methods for their species could have an idea of what values to expect.

Are sufficient details of methods and materials provided to allow replication by others?

Yes

Is the rationale for creating the dataset(s) clearly described?

Yes

Are the datasets clearly presented in a useable and accessible format?

Yes

Are the protocols appropriate and is the work technically sound?

Yes

Reviewer Expertise:

Genome assembly

I confirm that I have read this submission and believe that I have an appropriate level of expertise to confirm that it is of an acceptable scientific standard.

# Reviewer report: Reviewer response for version 1

**DOI:** 10.21956/wellcomeopenres.26133.r127271

**Published:** 2025-08-19

**Authors:** Hui Zhang

**Affiliations:** 1 Institute of Plant Protection, Fujian Academy of Agricultural Sciences, Fuzhou, Fujian, China

This manuscript by Barclay et al. presents the chromosomal-level genome assembly of *Cryptophagus acutangulus* , an acute-angled fungus beetle, contributing valuable genomic resources for Coleoptera. The authors provide comprehensive species background information and detailed methodologies covering specimen collection, sequencing, assembly, and quality assessments. The assembly metrics (e.g., BUSCO completeness: 98.4%; scaffold N50: 9.22 Mb) indicate a robust genome suitable for downstream analyses. I have no major concerns but note the following minor revisions to improve clarity and consistency:

 Please check and unify the inconsistent values between Abstract and Result sections (83.62% vs. 83.43% for chromosome scaffolding; 177.30 Mb vs. 177.32 Mb for genome size) to ensure data consistency throughout the manuscript.

 The phrase "the number of junior synonyms is greater the number of valid species" (Background section) is grammatically incorrect. It should be revised to "the number of junior synonyms is greater than the number of valid species".

 While optional, including preliminary gene annotations would further enhance the utility of this resource.

Are sufficient details of methods and materials provided to allow replication by others?

Yes

Is the rationale for creating the dataset(s) clearly described?

Yes

Are the datasets clearly presented in a useable and accessible format?

Yes

Are the protocols appropriate and is the work technically sound?

Yes

Reviewer Expertise:

genomics, transcriptomics, evolutionary biology

I confirm that I have read this submission and believe that I have an appropriate level of expertise to confirm that it is of an acceptable scientific standard.
